# Identification of key genes and regulators associated with carotenoid metabolism in apricot (*Prunus armeniaca*) fruit using weighted gene coexpression network analysis

**DOI:** 10.1186/s12864-019-6261-5

**Published:** 2019-11-20

**Authors:** Lina Zhang, Qiuyun Zhang, Wenhui Li, Shikui Zhang, Wanpeng Xi

**Affiliations:** 1 College of Horticulture and Landscape Architecture, Southwest University, Chongqing, 400716 People’s Republic of China; 2 Laboratory of Fruit Quality Biology, Zhejiang University, Zijingang Campus, Hangzhou, 310058 People’s Republic of China; 3 Agriculture National Fruit Tree Germplasm Repository, Xinjiang Academy of Agricultural Sciences, Luntai, Xinjiang 841600 People’s Republic of China

**Keywords:** Carotenoids, Apricot, Color, WGCNA, *Prunus armeniaca* L

## Abstract

**Background:**

Carotenoids are a class of terpenoid pigments that contribute to the color and nutritional value of many fruits. Their biosynthetic pathways have been well established in a number of plant species; however, many details of the regulatory mechanism controlling carotenoid metabolism remain to be elucidated. Apricot is one of the most carotenoid-rich fruits, making it a valuable system for investigating carotenoid metabolism. The purpose of this study was to identify key genes and regulators associated with carotenoid metabolism in apricot fruit based on transcriptome sequencing.

**Results:**

During fruit ripening in the apricot cultivar ‘Luntaixiaobaixing’ (LT), the total carotenoid content of the fruit decreased significantly, as did the levels of the carotenoids β-carotene, lutein and violaxanthin (*p* < 0.01). RNA sequencing (RNA-Seq) analysis of the fruit resulted in the identification of 44,754 unigenes and 6916 differentially expressed genes (DEGs) during ripening. Among these genes, 33,498 unigenes were annotated using public protein databases. Weighted gene coexpression network analysis (WGCNA) showed that two of the 13 identified modules (‘blue’ and ‘turquoise’) were highly correlated with carotenoid metabolism, and 33 structural genes from the carotenoid biosynthetic pathway were identified. Network visualization revealed 35 intramodular hub genes that putatively control carotenoid metabolism. The expression levels of these candidate genes were determined by quantitative real-time PCR analysis, which showed ripening-associated carotenoid accumulation. This analysis revealed that a range of genes (*NCED1*, *CCD1*/*4*, *PIF3/4*, *HY5*, *ERF003/5/12*, *RAP2–12*, *AP2*, AP2-like, *BZR1*, *MADS14*, *NAC2/25*, MYB1R1/44, *GLK1/2* and WRKY6/31/69) potentially affect apricot carotenoid metabolism during ripening. Based on deciphering the molecular mechanism involved in ripening, a network model of carotenoid metabolism in apricot fruit was proposed.

**Conclusions:**

Overall, our work provides new insights into the carotenoid metabolism of apricot and other species, which will facilitate future apricot functional studies and quality breeding through molecular design.

## Background

Carotenoids are widely distributed secondary metabolites that play important roles in plant physiology and are beneficial to human health as dietary components [1]. They confer flowers and fruits with yellow, orange and red colors, thereby helping to attract insects or animals to disperse seeds and pollen, and they are involved in photosynthesis and photoprotection. Carotenoids also scavenge free radicals in plants and help resist biotic and abiotic stresses [2]. In humans, dietary carotenoids provide vitamin A precursors, scavenge free radicals, strengthen immunity and prevent various diseases, such as certain cancers and cardiovascular diseases [3].

The carotenoid biosynthesis pathway has been well characterized in many plants [4]. The process starts with geranylgeranyl diphosphate (GGPP) synthesis, which involves a condensation reaction between isopentenyl diphosphate isomerase (IPP) and dimethylallyl diphosphate (DMAPP). GGPP is then converted into phytoene by phytoene synthase (PSY), which is in turn converted into lycopene via a series of desaturation and isomerization steps. Lycopene is cyclized by ε-cyclase (LCYE) and β-cyclase (LCYB) or by LCYB to produce α-carotene or β-carotene, respectively. These carotenes are further hydroxylated to produce zeaxanthin. Carotenoids can be cleaved into apocarotenoids (e.g., β-ionone, strigolactones, β-citraurin and abscisic acid) by carotenoid cleavage dioxygenase (CCD) and 9-cis-epoxycarotenoid dioxygenase (NCED) [5–7]. The associated structural genes in this biosynthetic pathway have been studied in detail in a number of plant species [1]. In tomato, the increased production of lycopene during the ripening-related fruit color change from green to red is caused by increased transcription of genes from early ‘upstream’ steps in the biosynthetic pathway, including *PSY*, phytoene desaturase (*PDS*), carotenoid isomerase (*CRTISO*) and deoxy-d-xylulose-5-phosphate synthase (*DXS*), and the downregulation of the ‘downstream genes’ *LCYB*, *LCYE*, and β-carotene hydroxylase (*CHYB*) [8–11]. Additionally, several transcription factors associated with carotenoid accumulation have been identified. For example, ethylene response factors (ERFs) and phytochrome interacting factors (PIFs) have been shown to reduce carotenoid accumulation by binding specifically to the *AtPSY* and *AtPDS* promoter in *Arabidopsis thaliana* [12, 13]. In tomato fruit tissues, the MADS box transcription factor ripening inhibitor (RIN) has been reported to regulate carotenoid accumulation by interacting with the *SlPSY1* promoter [14, 15]. However, many details of the regulatory network controlling carotenoid metabolism have yet to be identified.

Apricot (*Prunus armeniaca* L.) is an important fruit crop that is grown in temperate climates. Fresh apricots are an excellent source of diverse nutrients, including carotenoids, polyphenols, ascorbic acid and various microelements [16]. Processed apricot fruit byproducts have also been suggested to be valuable for human nutrition and the treatment of different diseases [16]. The ‘Luntaixiaobaixing’ (LT) apricot cultivar from China is commercially popular and valued by consumers due to its attractive flavor, shape and color. During ripening, its color changes from green to yellow and then to light yellow, and the fruit exhibits a reduction in carotenoid levels [17], which involves a range of carotenoid metabolic reactions, including synthesis, accumulation and cleavage. Thus, the ‘LT’ cultivar represents an excellent model for studying the regulatory mechanism of carotenoid metabolism.

RNA sequencing (RNA-Seq) is a powerful technology for studying gene expression, identifying genes and performing functional analysis [18], due to its high throughput, low cost, high accuracy and high sensitivity [19]. In recent years, RNA-Seq has been applied to the study of many fruit species to characterize the molecular processes underlying ripening and secondary metabolism [20–23]. It is a particularly effective approach for species for which whole-genome sequence data are not yet available, such as apricot. At present, the public resources of apricot transcriptome data are still very limited [24, 25].

In this study, we integrated weighted gene coexpression network analysis (WGCNA), gene-trait correlations and differential expression analysis in apricot fruit to identify key genes and regulators related to carotenoid metabolism during fruit ripening. Based on this analysis, a network model of carotenoid metabolism is proposed. These data will help advance the functional genomic analysis of carotenoid metabolism in apricot and other species.

## Results

### Changes in basic quality parameters and carotenoid content during apricot fruit ripening

**Fig. 1:**
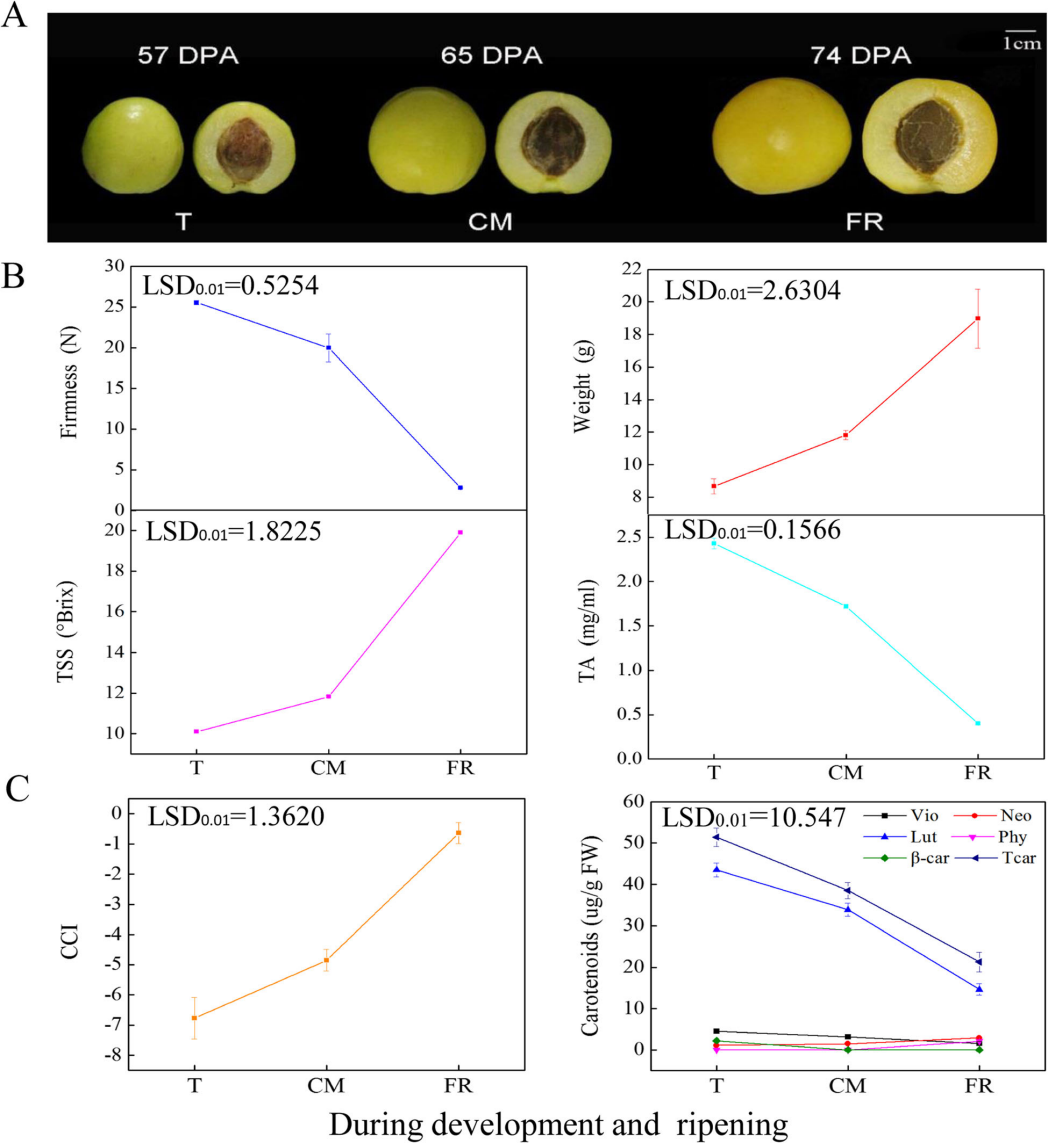
Characterization of fruit quality traits during ripening. **a** Color changes of apricot fruit. **b** Fruit firmness, fruit weight, total soluble solid (TSS) and titratable acid (TA) contents. **c** Changes in the citrus color index (CCI) and carotenoid content in apricot fruit. LSD, least significant difference (*p* < 0.01). Error bars represent ± SD of the means of three biological replicates

During the three apricot ripening stages (T, turning, 57 DPA (days post anthesis); CM, commercial maturation, 65 DPA; FR, fully ripe, 74 DPA), ‘LT’ fruit color changed from green to yellow and then from yellow to light yellow (Fig. 1a). Over the same period, fruit firmness dramatically decreased from 25.55 N to 2.77 N compression (*p* < 0.01), but fruit weight sharply increased from an average of 8.7 g to 19.0 g (Fig. 1b). Similarly, the content of fruit total soluble solids (TSS) significantly increased from 10.1 ^o^Brix to 19.9 ^o^Brix, while titratable acidity (TA) decreased substantially from 2.43 mg/mL to 0.4 mg/mL (*p* < 0.01) (Fig. 1b). We also observed that the citrus color index (CCI) values of the fruit significantly increased from − 6.77 to − 0.64 during ripening (*p* < 0.01) (Fig. 1c). Notably, the levels of lutein, violaxanthin and β-carotene significantly decreased during ripening (Fig. 1c), and the total carotenoid content markedly decreased from 51.4 μg/g fresh weight (FW) to 21.3 μg/g FW (*p* < 0.01). Conversely, the contents of neoxanthin and phytoene increased during the process (Fig. 1c). These results indicated that the ‘LT’ fruit underwent normal ripening based on a range of physiological and biochemical characteristics and that the cultivar is a ‘carotenoid loss’ type of cultivar.

### Transcriptome analysis of fruit ripening

**Fig. 2:**
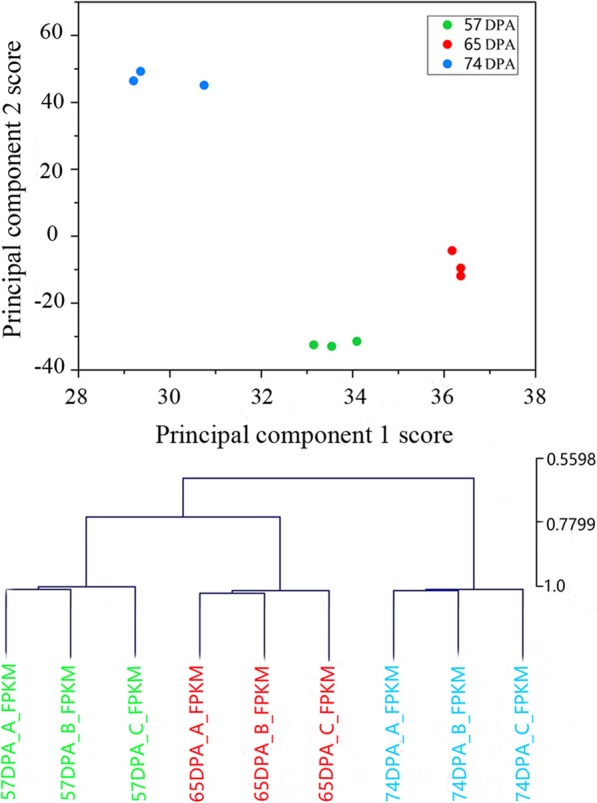
Principal component analysis (PCA) of transcriptome data. Three biological replicates per sample were analyzed for each ripening stage. The percentages on the axes indicate the values explained by each PCA. The green, red, and sky blue dots represent the transcriptomes of the samples obtained at 57 DPA (days post anthesis), 65 DPA and 74 DPA, respectively

Nine mRNA libraries were generated from the T, CM and FR stages of ‘LT’ fruit, with three biological replicates per stage. Average clean read numbers of 26.3, 26.0 and 27.2 million were derived from the T, CM and FR stages, respectively, corresponding to approximately 2.37, 2.34 and 2.45 gigabase pairs of nucleotides (nt), with GC percentages of 45.88, 45.58 and 46.16% (Additional file 1). Principal component analysis (PCA) confirmed the reproducibility of the sequence data for each stage (Fig. 2).

Next, a total of 44,754 unigenes with a mean length of 1196 bp and an N50 length of 1860 nt and a total of 71,243 contigs with a mean length of 529 bp and an N50 of 1303 nt, were assembled using Trinity software (Additional file 2). A total of 23,784 unigenes were assigned gene ontology (GO) terms and classified into 55 subcategories within the three standard categories of ‘biological process’, ‘cellular component’ and ‘molecular function’. The subcategories ‘metabolic process’ and ‘cellular process’ were the most highly enriched in the ‘biological process’ domain, while ‘cell’ and ‘cell part’ were the most highly enriched subcategories in the ‘cellular component’ domain, and ‘catalytic activity’ and ‘binding’ were the top ranked terms in the ‘molecular function’ domain (Additional file 3).

### Identification of differentially expressed genes (DEGs)

**Fig. 3:**
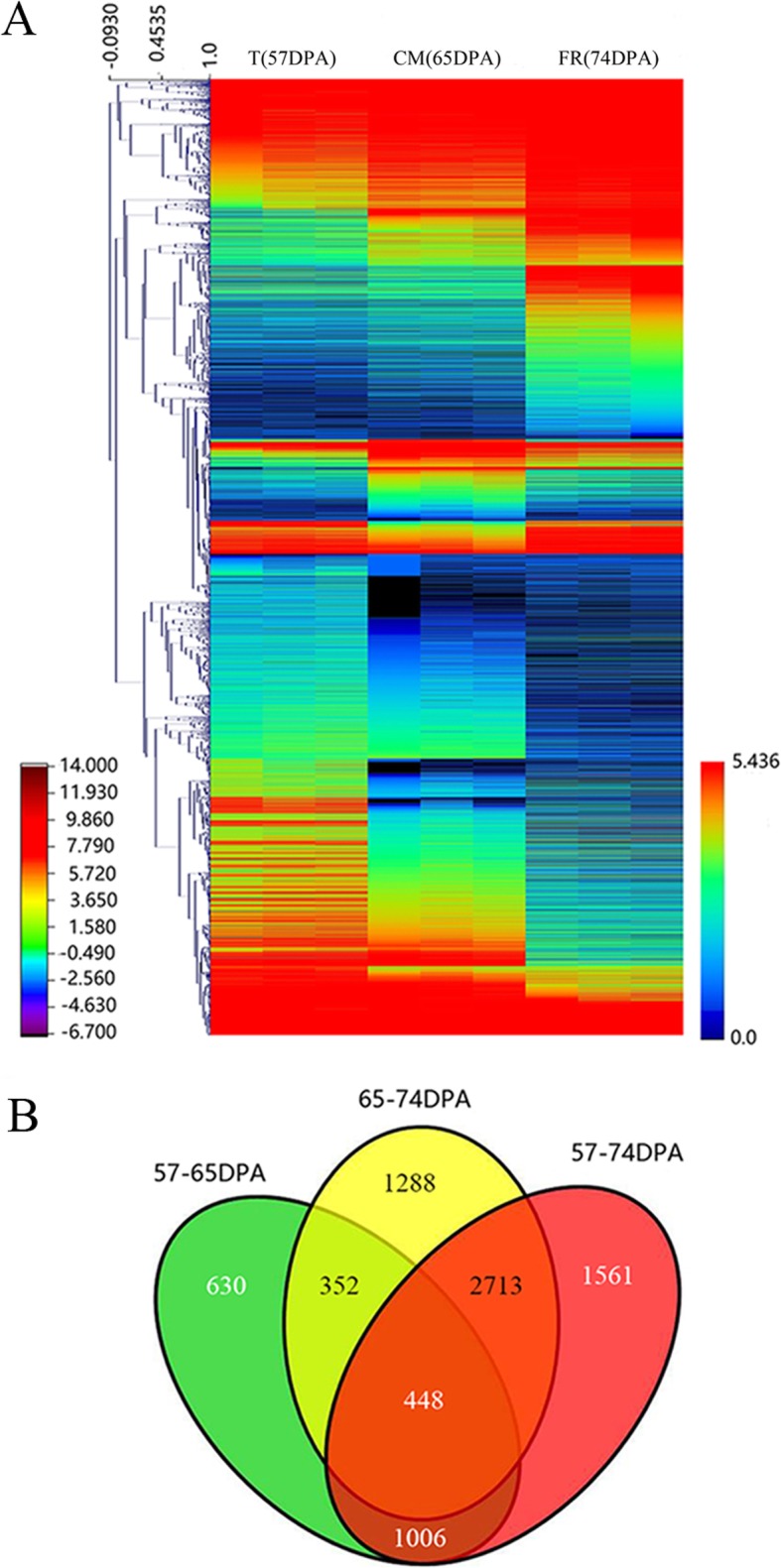
Expression profiles of differentially expressed genes (DEGs) during apricot fruit ripening. **a** DEG expression dynamics during fruit ripening. **b** Venn diagram indicates the number of DEGs in each of the three ripening stages

On the basis of the mapped reads, a total of 6916 DEGs were identified using fragments per kilobase of transcript per million mapped reads (FPKM) values, with 2436, 4801 and 5728 DEGs present in the T, CM and FR stages, respectively (Fig. 3a). The numbers of unique DEGs in the different stages were 630, 1288, and 1561 at 57 vs. 65 DPA, 65 vs .74 DPA and 57 vs. 74 DPA, respectively, and a total of 448 genes were expressed at all three stages. The identified DEGs were selected for further analysis (Fig. 3b and Additional file 4).

### GO enrichment and Kyoto encyclopedia of genes and genomes (KEGG) pathway analysis

To help identify the biological functions of the DEGs, the GO and KEGG databases were used to identify enriched terms/pathways at the three ripening stages for the identified DEGs. ‘Cellular process’, ‘metabolic process’ and ‘single organism process’ were the main categories in the ‘biological process’ domain, while the ‘cell’, ‘cell part’ and ‘organelle’ categories were enriched in the ‘cellular component’ domain. Finally, molecular functions such as ‘binding’, ‘catalytic activity’ and ‘transporter activity’ were the most highly enriched during the three ripening stages (Additional file 5).

KEGG pathway enrichment analysis was carried out to assess the biological significance of the DEGs during the three ripening stages. A total of 1773, 4719, 3215 unigenes from the T, CM and FR stages, respectively, were mapped to 20 KEGG pathways. ‘Metabolic pathways’, ‘plant-pathogen interaction’ and ‘plant hormone signal transduction’ were the most significantly enriched metabolic pathways. We identified 19, 33 and 16 DEGs from the carotenoid biosynthetic pathway enriched in the T, CM and FR stages, respectively. This analysis suggested that the three enriched metabolic pathways may play a role in carotenoid metabolism in apricot fruit (Additional file 6).

### Weighted gene coexpression network analysis (WGCNA)

**Fig. 4:**
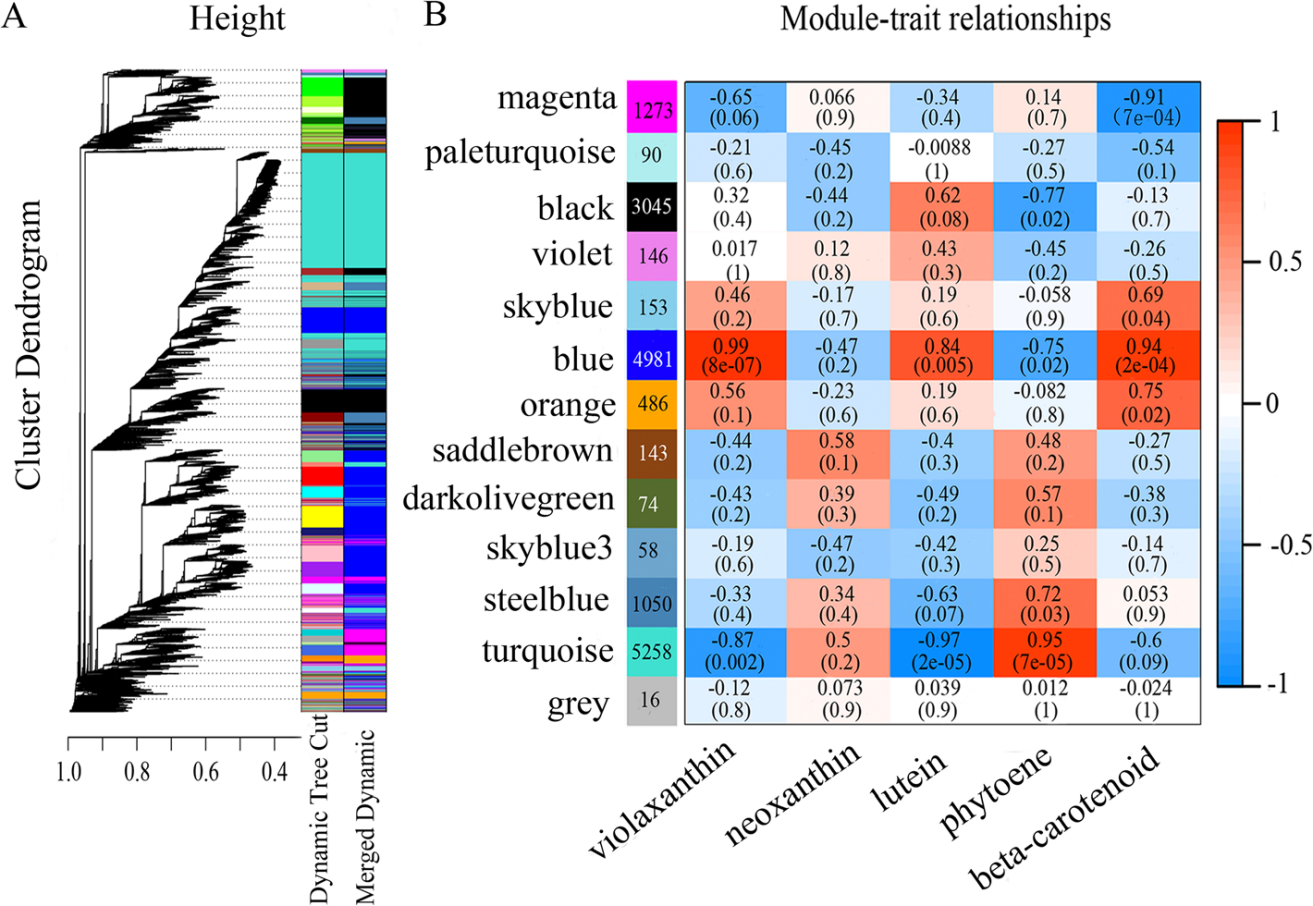
Weighted gene coexpression network analysis (WGCNA) of apricot fruit during ripening. **a** Hierarchical clustering showing 13 modules of coexpressed genes. Each leaf in the tree represents one gene. **b** Module-carotenoid correlations and corresponding *p*-values. The left panel shows the 13 modules and the number of genes in each module. The right panel shows a color scale for module/trait correlations from − 1 to 1

WGCNA is a method for describing the correlations in patterns of gene expression and for revealing clusters, or modules, of genes whose expression is highly correlated. This method allows the identification of module eigengenes and intramodular hub genes and the association of modules with one another and with sample traits [26]. Here, 13 coexpression modules were identified by WGCNA, among which the ‘blue’ and ‘turquoise’ modules were significantly associated with carotenoid metabolism during ripening (Fig. 4). Analysis of the module-trait relationships suggested that the ‘blue’ module, containing 4981 genes, was highly positively correlated with levels of violaxanthin (*r* = 0.99, *P* = 8e-07), lutein (*r* = 0.84, *P* = 0.005) and β-carotene (*r* = 0.94, *P* = 2e-04) and negatively correlated with neoxanthin (*r* = − 0.47, *P* = 0.2) and phytoene (*r* = − 0.75, *P* = 0.02) contents during ripening. The abundance of phytoene was highly positively correlated with gene expression in the ‘turquoise’ module, which contained 5258 genes, with a coefficient of 0.95 (*P* = 7e-05), but the levels of violaxanthin and lutein were significantly negatively correlated with gene expression, with coefficients of − 0.87 (*P* = 0.002) and − 0.97 (*P* = 2e-05), respectively (Fig. 4b). These results suggested that the genes in these two modules were associated with carotenoid accumulation.

### DEGs involved in carotenoid biosynthesis

**Fig. 5:**
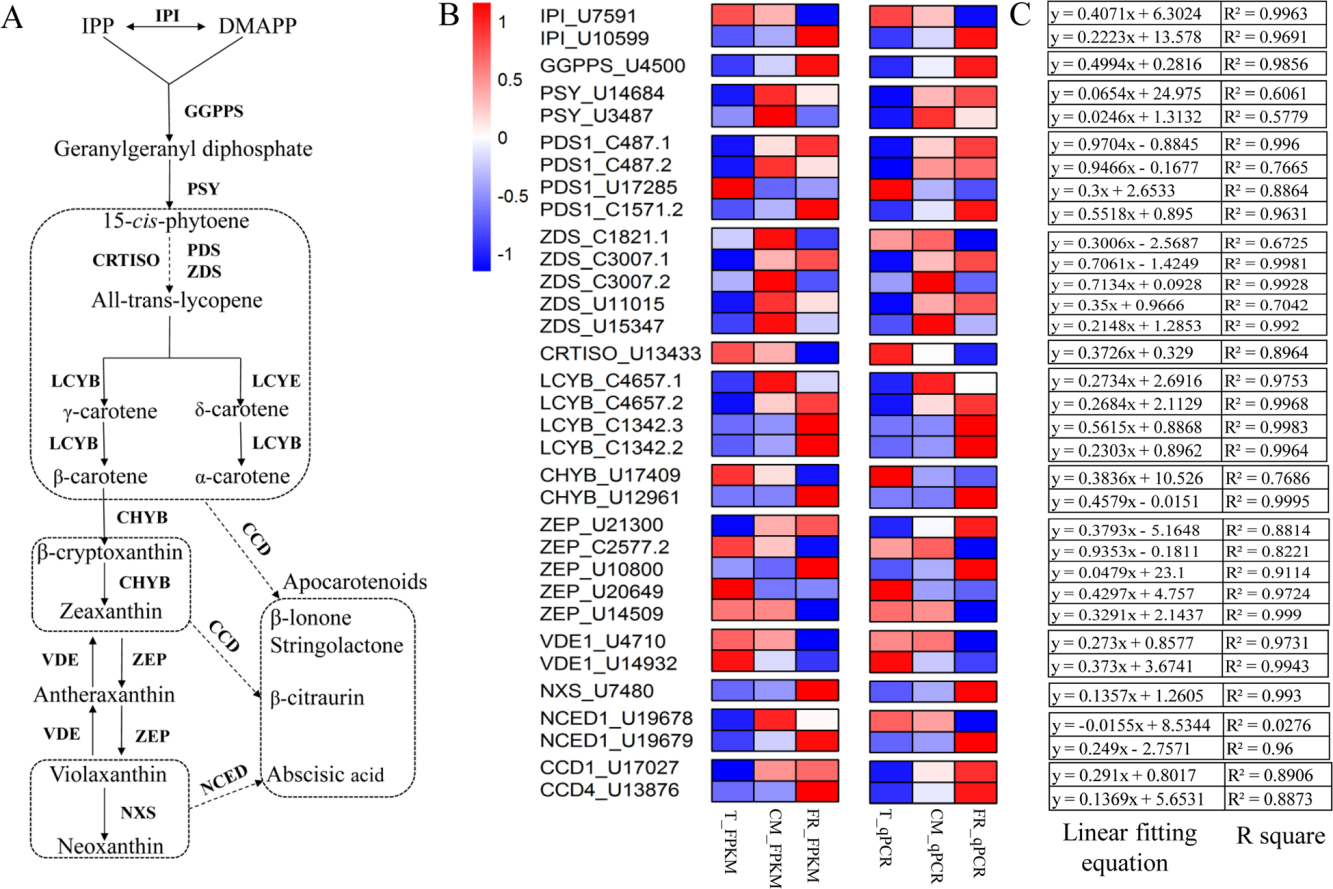
Expression of genes related to carotenoid biosynthesis in apricot fruit. **a** Carotenoid biosynthesis pathway in apricot fruit. IPI, isopentenyl diphosphate isomerase; GGPPS, geranylgeranyl diphosphate synthase; PSY, phytoene synthase; PDS, phytoene desaturase; ZDS, ζ-carotene desaturase; CRTISO, carotenoid isomerase; LCYE, lycopene ε-cyclase; LCYB, lycopene β-cyclase; CHYB, β-carotene hydroxylase; ZEP, zeaxanthin epoxidase; VDE, violaxanthin de-epoxidase; NXS, neoxanthin synthase; CCD, carotenoid cleavage dioxygenase; NCED, 9-cis-epoxycarotenoid dioxygenase. **b** Heatmap of the expression of genes related to carotenoid biosynthesis during fruit ripening. Columns and rows represent samples and gene names in the heatmap, respectively. Red, blue and white indicate high expression, low expression and the absence (or undetectable levels) of detectable transcripts at the corresponding stage, respectively. **c** The linear fitting equation and R squared values between the FPKM and qRT-PCR values

In the carotenoid biosynthesis pathway (Fig. 5a), we identified 33 DEGs that were annotated as functioning in carotenoid metabolism; their expression levels were represented by both FPKM and qPCR values (Fig. 5b, Additional file 7), and good linear positive correlations were observed between them (Fig. 5c). During ripening, the expression levels of two *IPI* genes (U7591 and U10599) were downregulated and upregulated, respectively. In addition, the expression of the geranylgeranyl diphosphate synthase (*GGPPS*) gene was significantly upregulated, and the expression levels of two *PSY* (U14684 and U3487) genes were upregulated and up/downregulated, respectively. The expression levels of four *PDS1* genes gradually increased during ripening, with *PDS1* (U17285) representing the exception. Three ζ-carotene desaturase (*ZDS*) genes (CL1821.1, CL3007.2 and U15347) displayed similar up/downregulated expression patterns, and other *ZDS* genes showed significant upregulation. *CRTISO* expression markedly decreased during ripening, and the expression of three *LCYB* genes showed similarly upregulated expression, with *LYCB* (CL4657.1) representing the exception, but the expression of two *CHYB* genes showed the opposite expression pattern. Five zeaxanthin epoxidase (*ZEP*) genes exhibited different levels of expression. Two violaxanthinde-epoxidase 1 (*VDE1*) genes were downregulated, while the expression of neoxanthin synthase (*NXS*) was upregulated at the FR stage. The expression of the *NCED1* gene as well as *CCD1* and *CCD4* gradually increased during ripening. Thus, numerous DEGs that were involved in the ripening-related synthesis and accumulation of carotenoids were identified.

### Visualization of gene networks

**Fig. 6:**
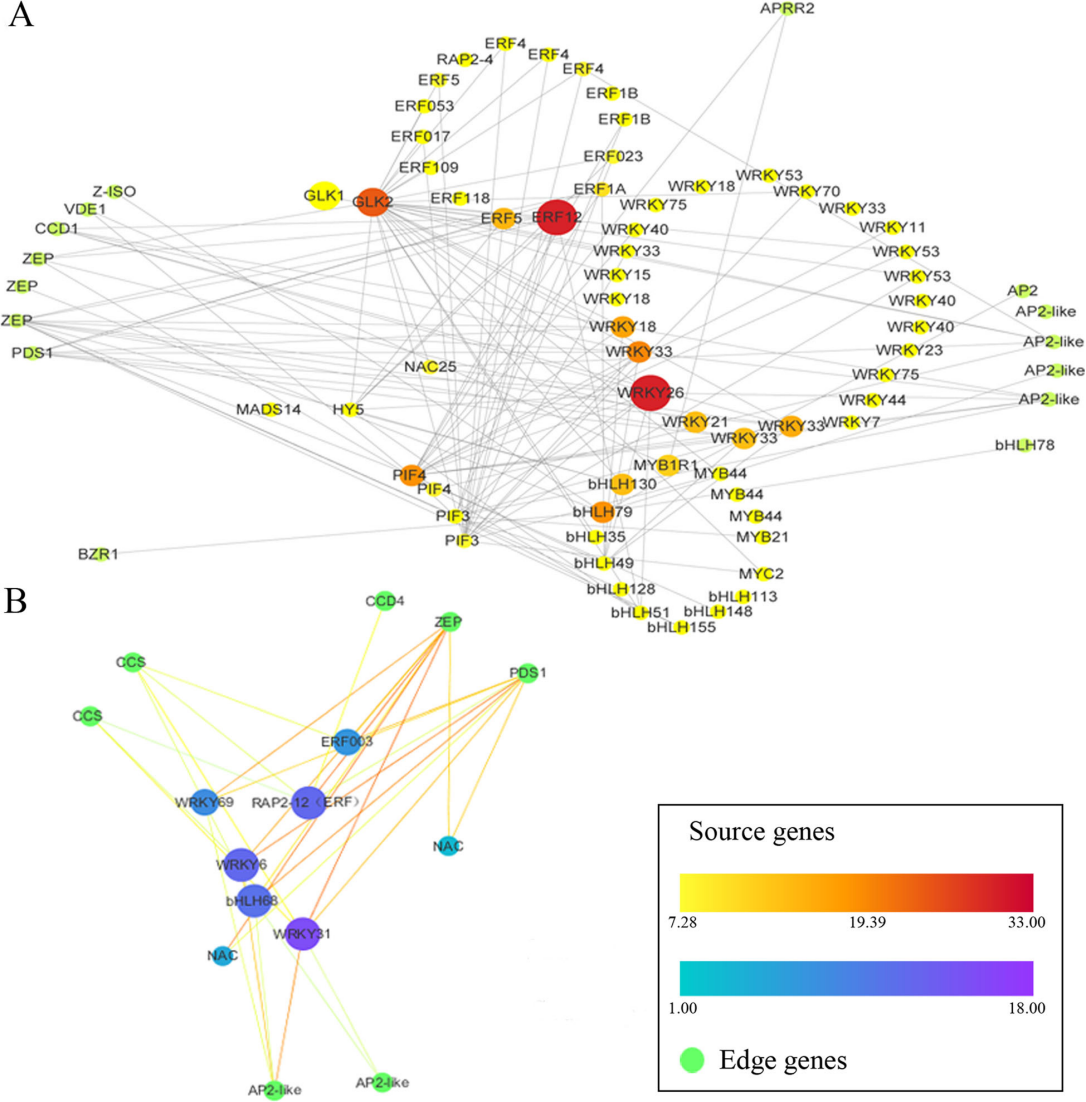
Coexpression networks of transcription factors and structural genes involved in carotenoid metabolism. The network includes transcription factors and structural genes from the ‘blue’ (**a)** and ‘turquoise’ (**b**) modules. Dot sizes and colors represent the numbers of connections between genes

A total of 78 genes in the ‘blue’ module and 15 genes in the ‘turquoise’ module were analyzed using Cytoscape version 2.8. Both modules exhibited a significant association with carotenoid contents and gene expression of > 0.4 (Fig. 6 and Additional file 8). In the two networks, 15 *ERF*, 9 *bHLH* and 24 *WRKY* family members were identified as intramodular hub genes and were therefore putatively associated with the regulation of carotenoid metabolism. The carotenoid biosynthesis pathway genes *PDS1*, *CCD1*, *CCD4*, *VDE1* and *ZEP* were identified as key control points for carotenoid metabolism (Fig. 6a and b). In addition to these structural genes, we identified transcription factors in the two modules, including U7919, C4195.1 and U6922, which are homologs of the *Arabidopsis thaliana* MYB1R1, MYB21 and MYB44 proteins, respectively, involved in the regulation of carotenoid metabolism. Homologs of the *A. thaliana* transcription factors MYC2, GLK1, GLK2, NAC25, HY5, PIF3, MADS14, WRKY6, WRKY31, WRKY69, ERF003, RAP2–12 (ERF), NAC and bHLH68 were also identified.

### Expression of candidate genes and regulators during fruit ripening

**Fig. 7:**
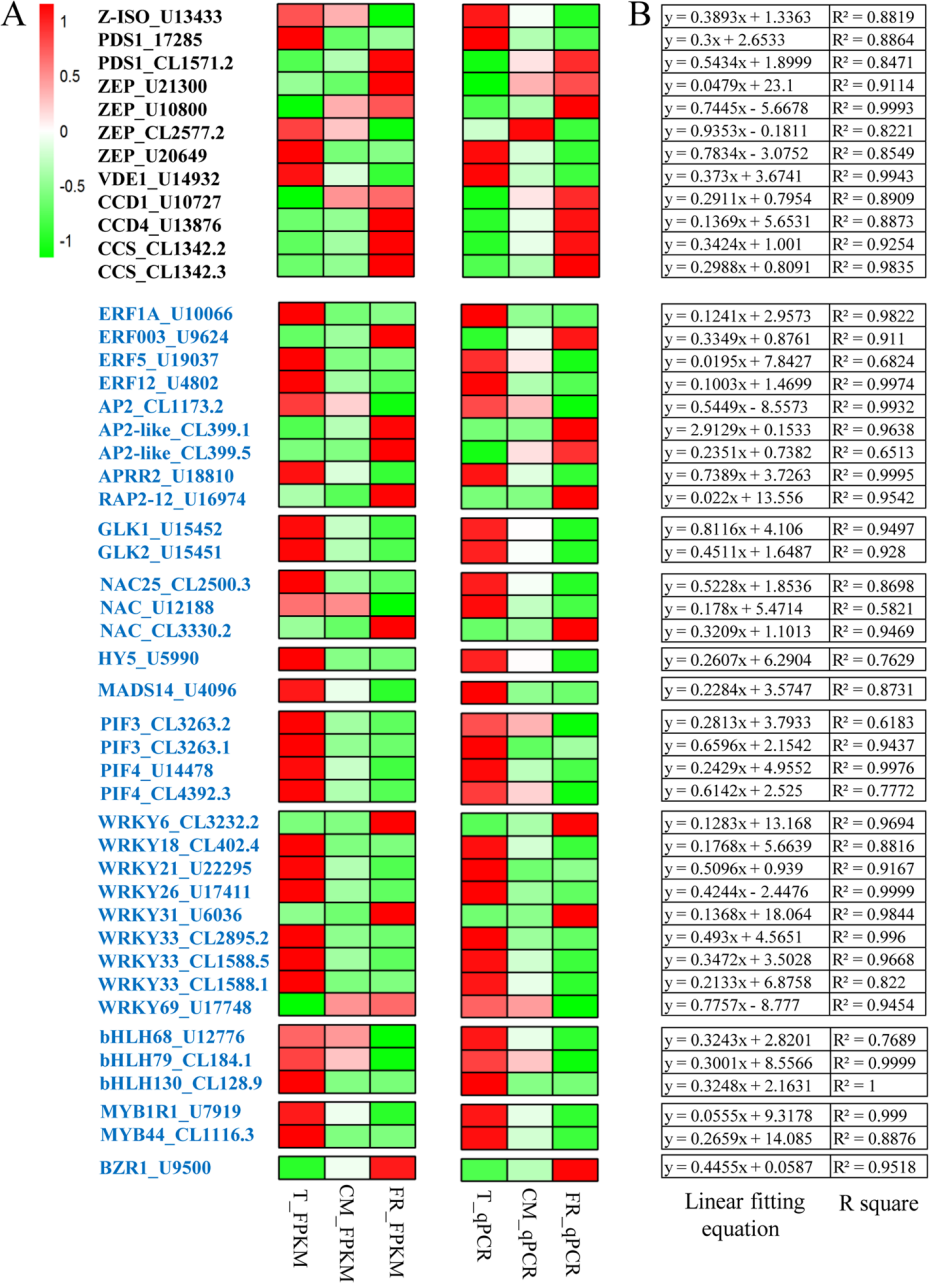
The expression of genes and regulators related to carotenoid metabolism in the visualized networks for the ‘blue’ and ‘turquoise’ modules. **a** Heatmap of the expression of structural genes related to carotenoid biosynthesis during fruit ripening. Columns and rows in the heat map represent the developmental stage and gene name, respectively. The black and blue genes represent structural genes and transcription factors, respectively. Red, green and white indicate high expression, low expression and the absence (or undetectable levels) of detectable transcripts at the corresponding stage, respectively. **b** The linear fitting equation and R squared values between the FPKM and qRT-PCR values

To verify the reliability of the RNA-Seq results and ascertain the relationship between candidate genes and carotenoid metabolism, we also measured the expression levels of the 12 candidate structural genes and 35 transcription factors by qRT-PCR during fruit ripening (Fig. 7a). Overall, all upstream genes related to carotenoid biosynthesis, such as *PDS1* (CL1571.2), *ZEP* (U21300 and U10800), *CCD*1/4 and capsanthin/capsorubin synthase (*CCS*), were upregulated in the ‘LT’ fruit, while the expression levels of 15-cis-zeta-carotene isomerase (*Z-ISO*), *PDS1* (U17285), *ZEP* (U20649) and *VDE1* were downregulated. *ERF*003, *AP2-like, RAP*2–12, *NAC* (CL3330.2), *WRKY*6/31 and *BZR*1 were significantly upregulated during ripening, but other genes showed opposite expression patterns. Linear regression analysis demonstrated that the FPKM values were highly correlated with the qRT-PCR results (Fig. 7b).

## Discussion

**Fig. 8:**
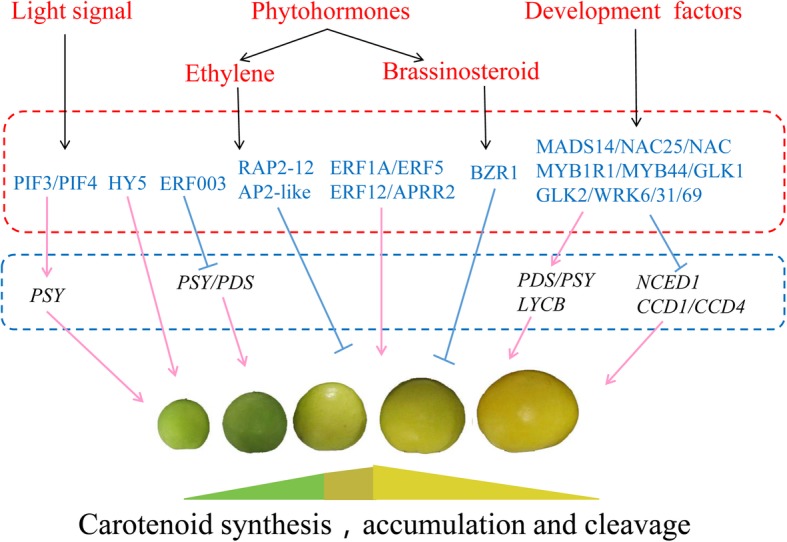
The proposed model of carotenoid metabolism in apricot during fruit ripening. Pink arrows represent positive regulation; blue lines represent negative regulation; black and skyblue genes represent structural genes and transcription factors, respectively. PIF, phytochrome interacting factor; HY5, LONG HYPOCOTYL5; RAP2–12, Ethylene-responsive transcription factor RAP2–12; ERF, Ethylene response factor; BZR1, BRASSINAZOLE RESISTANT1; MADS14, MADS-box transcription factor 14; NAC, NAC transcription factor; MYB, MYB transcription factor; GLK, GOLDEN 2-LIKE transcription factor

An increasing number of studies highlight the complexity and diversity of carotenoid metabolism in different plant species [1, 4]. Here, we used the apricot cultivar ‘LT’, which exhibits a specific carotenoid accumulation pattern that includes biosynthesis, sequestration and degradation during fruit ripening, to identify key candidate genes and regulators related to carotenoid metabolism by WGCNA. Based on our results, we conclude that carotenoid metabolism in apricot fruit is cocontrolled by light signals, phytohormones, and developmental factors, and a network regulatory model was proposed (Fig. 8).

Light is a key factor in plant growth and development, since it provides energy for photosynthesis and regulates photomorphogenesis as a signal [4, 5]. PIF is a member of the bHLH transcription factor family and can integrate multiple environmental and intercellular signals to control growth and development, stress responses and secondary metabolism. A light signal causes a rapid transfer of active phytochrome to the nucleus, where it interacts with PIFs [27, 28]. In the present study, we observed that the expression of two *PIF3* genes and two *PIF4* genes was positively correlated with carotenoid accumulation during ripening (Fig. 1c and Fig. 7a). Our results are in agreement with the observation that PIF5 functions as a positive regulator of the MEP pathway and increases the accumulation of chlorophylls and carotenoids in cultured cells [29], but they contrast with the finding that PIF1 proteins bind directly to the G-box element in the *PSY* promoter to inhibit its expression, resulting in reduced carotenoid accumulation [13], which suggests that PIF gene members may play a different role in carotenoid metabolism. Two light signaling transcription factors, LONG HYPOCOTYL5 (HY5) and COP1-like, were previously identified in tomato fruit. In contrast to *LeCOP1LIKE*, repression of *LeHY5* results in a decrease in carotenoid content [30]. In this study, we observed that the expression level of *HY5* gradually decreased during ripening, in parallel with the decline in carotenoid levels in apricot fruit. In *Arabidopsis*, HY5 and PIFs form a dynamic activation-suppression transcriptional module that is responsive to light and temperature cues and regulates carotenoid and chlorophyll accumulation by direct binding to the same G-box cis element of a target gene [31]. However, more work is required to confirm whether this fine-tuning module exists in fruit. Taken together, these results suggest that light signaling-associated transcription factors are involved in regulating carotenoid accumulation in ripening apricot fruit.

Previous studies have shown that the synthesis and accumulation of carotenoids are regulated by various phytohormones. Tomato fruit ripening is triggered by an increase in ethylene biosynthesis and is accompanied by a large increase in β-carotene and lycopene content as well as the expression of *SIPSY1* and *SIPDS*, which is ethylene dependent [32]. Similarly, ethylene induces the expression of *PSY1* and *PDS* and promotes the accumulation of the corresponding carotenoid product in apricot [32]. This is consistent with our observation that the upregulation of the expression of *PDS1* (CL1571.2) and the ethylene response factor *ERF003* was correlated with increased phytoene and neoxanthin levels during ripening. We propose that *PDS1* may also be regulated by ethylene in apricots to promote carotenoid accumulation (Fig. 1c and Fig. 7). In tomato, the suppression of *SIAP2a* mediated by RNAi results in lower levels of carotenoids, and reduced expression of *SIERF6* enhances carotenoid accumulation, suggesting that these genes play positive and negative roles, respectively, in carotenoid accumulation during ripening [33, 34]. In our study, ERF genes showed two distinct expression patterns, indicating that ethylene signaling member has different functions in the regulation of carotenoid accumulation in apricot fruit (Fig. 7a). In *A. thaliana*, the ERF gene *RAP2.2* directly binds to the *AtPSY* and *AtPDS* promoters to reduce carotenoid accumulation [12]. Here, we also observed that the increased expression levels of *RAP2–12* showed a negative correlation with the levels of carotenoid accumulation, while *ERF1A, ERF5/12*, and *APRR2* were positively correlated with carotenoid accumulation (Fig. 7a).

In other studies, the application of the brassinosteroid (BR) 2,4-epibrassinolide (EBR) to tomato has led to increased levels of lycopene and a reduced chlorophyll content in the fruit [35]. Additionally, the transcription factor BRASSINAZOLE RESISTANT1 (*BZR1*) has been shown to play a role in carotenoid accumulation in tomato fruit [36]. In this study, we observed increased expression of *BZR1* during ripening, suggesting that the BR signaling pathway may also be associated with carotenoid accumulation (Fig. 7b). Over time, our results are consistent with the regulation of carotenoid accumulation during apricot fruit ripening by both ethylene and BR.

Numerous developmental regulatory factors have been shown to affect the synthesis and accumulation of carotenoids. These include the MADS-box genes *AGAMOUS-LIKE 1* and *FRUITFUL1*, which modulate carotenoid accumulation during tomato fruit ripening [37], and the MADS-box transcription factors RIN and FUL1/TDR4, which directly bind to the *SlPSY1* and *SIPDS1* promoters to regulate carotenoid synthesis [38, 39]. Recently, a sweet orange (*Citrus sinensis*) transcription factor, CsMADS6, was found to promote carotenoid accumulation by coordinately modulating the expression of *LCYB1*, *PSY*, *PDS* and *CCD1* [40]. Here, we observed that the downregulation of *MADS14* expression was accompanied by upregulated expression of *CCD1/4* and downregulated expression of *PDS1* (U17285), consistent with their involvement in carotenoid accumulation during ripening (Fig. 1c and Fig. 7a). NAC transcription factors are plant specific and play important roles in the development of various organs and fruit [41]. Overexpression of *SlNAC1* and *SlNAC4* results in reduced carotenoid levels and inhibition of fruit ripening by altering the carotenoid pathway flux and decreasing the levels of ethylene biosynthesis genes belonging to system-2, but a newly identified tomato NAC (N AM/A TAF1/2/C UC2), SlNAC4, functions as a positive regulator of fruit ripening and carotenoid accumulation [41, 42]. In this study, we observed that three NAC (*NAC25, NAC_U12188*, *NAC_CL3330.2*) genes with different expression patterns were involved in regulating carotenoid accumulation (Fig. 7a).

MYB TFs regulate diverse plant developmental processes. The R2R3-MYB TF RCP1 is a master regulator that controls the entire CBP during flower development and positively regulates carotenoid biosynthesis during *Mimulus lewisii* flower development [43]. MYB7 can activate the *AdLCYB* promoter, thereby modulating carotenoid pigment accumulation in kiwifruit (*Actinidia deliciosa*) [44]. Similarly, the significantly reduced expression of *MYB1R1* and *MYB44* during ripening suggested their involvement in carotenoid accumulation in apricots (Fig. 7a). In contrast, CrMYB68 suppresses the expression of *CrBCH2* and *CrNCED5* in the citrus carotenoid pathway [45]. Another MYB transcription factor, GOLDEN2-LIKE (GLK2), has been shown to increase lycopene levels in tomato fruit at the red ripe stage [46–48]. Here, the expression levels of *GLK1* and *GLK2* decreased during ripening, suggesting that their expression may limit carotenoid biosynthesis during ripening in apricots (Fig. 7a). Overexpression of *SlPRE2*, an atypical bHLH transcription factor, negatively affects plant morphology and fruit pigment accumulation in tomato by downregulating the expression of the chlorophyll-related genes *GLK2* and RbcS and the carotenoid biosynthesis-related genes *PSY1A* and *ZDS* in ripening tomato [49], suggesting that bHLH TFs may regulate carotenoids upstream of GLKs. In the present study, we identified three bHLHs with downregulated expression patterns, but their regulatory roles are still unknown. We also examined the expression patterns of the WRKY6/31/69 transcription factors (Fig. 7b), whose homolog OfWRKY3 has been implicated as a positive regulator of the *OfCCD4* gene in *Osmanthus fragrans* [50]. Collectively, these results indicated that many developmental factors take part in regulating carotenoid biosynthesis and accumulation in apricots.

We noted that higher expression levels of *PDS1*, *ZEP* and *VDE1* were detected in ‘LT’ apricot fruit. In addition*,* the two genes responsible for carotenoid cleavage (*CCD1* and *CCD4*) exhibited remarkable upregulated expression patterns in the ‘carotenoid loss’ type of cultivar during fruit ripening (Fig. 7a). Similarly, studies in strawberry showed that changes in lutein content were closely related to the expression level of *CCD1* during ripening [51]. *CCD4* has also been shown to control peach flesh pigmentation, with high *CCD4* transcript abundance being observed in white flesh peach, which is associated with the emission of carotenoid-derived volatiles [52]. In this study, the upregulated expression of two *ZEP* genes (U10800 and U21300) was accompanied by a decrease in violaxanthin during ripening. Loss of function of *ZEP* in the *aba1* mutant of *A. thaliana* and the *aba2* mutant of tobacco causes the accumulation of high zeaxanthin levels in leaves [53, 54]. These observations suggest that *ZEP*, *CCD1* and *CCD4* play central roles in determining carotenoid levels in apricots and that degradation is the key control point for carotenoid accumulation.

## Conclusions

In this study, WGCNA analysis revealed that two modules (‘blue’ and ‘turquoise’) are highly correlated with carotenoid metabolism. Structural genes *(CCD1*, *CCD4*, *VDE1*, *ZEP* and *PDS1*) are associated with the carotenoid biosynthesis pathway, and transcription factors related to light signaling (PIF3/4 and HY5), phytohormones (ERF4/5/12, AP2, AP2-like and BZR1) and development factors (MADS14, NAC2/25, MYB1R1/44, GLK1/2, and WRKY6/31/69) may play important regulatory roles in carotenoid metabolism, but the specific regulatory mechanism still needs to be elucidated. Our findings not only provide new insights into the mechanisms of carotenoid metabolism, but these datasets also provide a useful platform for further functional studies of candidate genes.

## Methods

### Plant materials

From May to July 2017, fruits from the ‘LT’ cultivar were harvested at different ripening stages (turning, T, 57 DPA (days post anthesis); commercial maturation, CM, 65 DPA and fully ripe, FR, 74 DPA) from the National Fruit Tree Germplasm Repository, Academy of Xinjiang Agricultural Sciences, Luntai, Xinjiang, China (Fig. 1a). All samples were transported to the laboratory on the day of harvest. A set of fruit of uniform size and with no obvious mechanical damage was selected for the subsequent experiments. Fifty fruits were used as one replicate, and three biological replicates were used for each sample. For each replicate, 20 fruit were used to measure a range of physiological indices. Other fruit were cut into small cubes and then immediately frozen in liquid nitrogen and stored at − 80 °C for measurement of carotenoid contents and for RNA-Seq and gene expression analysis.

### Determination of basic quality parameters

Flesh firmness was measured using a firmness tester (Model: HL-300, Xianlin Non Detection Device Co., Ltd., Nanjing, China) with an 8 mm probe. To determine TSS values, the juice was squeezed from three fruits per replicate, pooled and analyzed using a handheld digital refractometer (B32T Brix Meter, Guangzhou Ruiqi Trading Company, Guangdong, China). TA values were determined after the juice sample was diluted 100 times with pure water. The fruit color parameters were measured using a Hunter’s Mini Scanning Colorimeter (Hunter Associates Laboratory, Inc., Reston, VA, USA). The CCI was calculated with the following formula: CCI = 1000 × a*/(L* × b*), using three fruits as a single replicate and three biological replicates.

### Extraction and identification of carotenoids

Carotenoids were extracted as previously described with some modifications [55]. Eight grams of fruit peel was placed in a screw-top tube, and 50 mL of extraction solvent (hexane/acetone/ethanol, 50:25:25, v/v) was added. After standing for 30 min, the samples were centrifuged for 5 min at 6500 rpm. The colored top layer of hexane was recovered and transferred to a volumetric flask, where it was dried under nitrogen, and dissolved in 2 mL of methyl tert-butyl ether (MTBE) before being combined with 2 mL of 10% methanol/potassium hydroxide. The mixture was allowed to stand for 1 h and separated in a separating funnel and was rinsed twice with water and once with 0.1% butylhydroxytoluene (BHT)/MTBE. The rinsed solution was transferred to a brown bottle and dried under nitrogen before 2 mL methanol/acetone (2,1) was added, and the solution was filtered through a 0.22 m filter membrane.

Carotenoids were identified by high-pressure liquid chromatography (HPLC, Waters, Milford, MA, USA) with a C30 chromatography column (250 mm × 4.6 mm, 5 μm, YMC, Wilmington, NC, USA). The mobile phase flow rate was 1 mL/min, and the column temperature was 25 °C, with a detection wavelength of 450 nm and an injection volume of 20 μL. The mobile phase composition was as follows: methanol, MTBE and water. Carotenoids were identified by comparison to standard retention times and UV-visible spectral peaks. The quantification of carotenoids was performed using a standard curve and expressed as μg/g FW. Three biological replicates for each sample were used.

### RNA extraction, library construction and data analysis

Total RNA was isolated and extracted from 1 g of fruit peel for each sample using a Tiangen reagent kit (Tiangen, Beijing, China). RNA purity, concentration and integrity were determined using a Nanodrop 2000 (NanoDrop 2000, Wilmington, DC, USA) and denaturing agarose gel electrophoresis. ‘LT’ RNA-Seq libraries of T-, CM- and FR-stage fruit were constructed using an Illumina TruSeq RNA Library Prep Kit v2 following standard procedures, and three biological replicates were used for each stage. The libraries were sequenced using an Illumina HiSeq™ 2000 system at the Beijing Genomics Institute (BGI), China. Clean reads were obtained by removing the linker, repetitive, redundant and low-quality sequences from the raw reads. The sequence data from this study have been deposited in the National Center for Biotechnology Information (NCBI) under Sequence Read Archive (SRA) accession number PRJNA530709.

### De novo assembly and functional annotation

De novo *transcriptome* assembly was carried out using the short read assembly program Trinity (http://trinityrnaseq.sourceforge.net/) [56]. The assembled unigenes were first filtered to remove redundant sequences and further spliced using the TGICL (https://sourceforge.net/projects/tgicl/files/tgicl%20v2.1/) version 2.1 software package [57]. The sequences were then clustered into homologous transcripts to obtain the final unigenes. Subsequently, the unigenes were divided into two classes: one with several unigenes with > 70% similarity, with the prefix CL followed by the cluster ID, and the other consisting of singletons, with the prefix unigene. Finally, BLASTx alignment (e-value < 0.00001) was performed between the unigenes and the NR, Swiss-Prot, KEGG and COG protein databases, and the best alignments were used to determine the sequence direction of the unigenes. The GO annotations of unigenes were obtained using Blast2GO Version 2.3.4 software (https://www.blast2go.com) [58]. WEGO Version 2.0 (Web Gene Ontology Annotation Plot) software (http://wego.genomics.org.cn) was used to provide the GO functional classifications of all the unigenes [59].

### Principal component analysis (PCA) and identification of DEGs

PCA was performed to verify the reproducibility of the sequence data using a previously described method [60]. DEGs between each sample were identified on the basis of FPKM values [61] and pairwise comparisons, with a false discovery rate (FDR) threshold of < 0.001 and an absolute value of the log2 Ratio ≥ 1 using the DESeq R package (v1.10.1) [62]. The DEGs were also analyzed by heatmap clustering using Origin Pro 2018 (Origin Lab, Northampton, MA, USA).

### GO and KEGG pathway enrichment analysis

The DEGs were mapped to each term in the GO database (http://www.geneontology.org/) [63], and the number of genes associated with each term was determined. Based on the gene numbers for each GO term, a hypergeometric test was applied to determine the GO terms that were significantly enriched for DEGs compared with the genome background. A corrected *p*-value < 0.05 was used as the threshold, and the GO terms meeting this condition were defined as significantly enriched for DEGs. A KEGG pathway enrichment analysis was performed using the KEGG database (http://www.genome.jp/kegg/) [64]. After multiple tests and corrections, we determined that pathways with a Q-value 0.05 were significantly enriched in DEGs.

### WGCNA and visualization of gene networks

A total of 16,773 unigenes with FPKM values > 1 were used to perform WGCNA by using the WGCNA package in R [26]. The modules were obtained using the automatic network construction function. Modules were identified using default settings, except that the soft power was 10, the min module size was 30, and the merge cut height was 0.25. Finally, Cytoscape version 2.8 (http://www.cytoscape.org/) was used to visualize the carotenoid metabolism regulatory network based on the WGCNA modules [65].

### Real-time quantitative PCR

The expression levels of candidate structural genes and transcription factors were measured in ‘LT’ fruit at three ripening stages by qRT-PCR analysis. Ribosomal RNA and actin gene expression was used as a normalization reference as previously described [24]. Specific primers were designed using Primer5 (Additional file 9). Gene expression levels were detected using an iQ5 instrument (Bio-Rad Laboratories, Inc. America) with the SYBR® Premix Ex TaqTM II Kit (TaKaRa Biotechnology (Dalian) Co, Ltd., China). The amplification program was as follows: 95 °C for 1 min, followed by 40 cycles at 95 °C for 20 s, 58 °C for 20 s and 72 °C for 30 s. Each qRT-PCR analysis was performed in triplicate, and the mean value was used for the qRT-PCR analysis. The relative expression of the genes was calculated according to the 2 ^–ΔΔCT^ method [66], and OriginPro 2018 (OriginLab, Northampton, MA, USA) was used to analyze the data.

## Supplementary information

**Additional file 1.** Throughput and quality of RNA-Seq data in apricot fruit.

**Additional file 2.** Statistics of assembly quality.

**Additional file 3.** Gene ontology (GO) classification map. The horizontal axis represents the type of GO function; the vertical axis on the right represents the number of unigenes annotated to the corresponding GO function; and the vertical axis on the left represents the percentage of the number of unigenes within the total.

**Additional file 4.** Differentially expressed genes (DEGs) shown in Fig. 3b.

**Additional file 5.** Gene ontology (GO) annotation of differentially expressed genes (DEGs). (A) T vs CM, (B) T vs FR, (C) CM vs FR. The unigenes were annotated in three categories: biological process, cellular component and molecular functions. The right y-axis indicates the number of genes in a category, and the left y-axis indicates the percentage of a specific gene.

**Additional file 6.** Significantly enriched differentially expressed genes (DEGs) in Kyoto Encyclopedia of Genes and Genomes (KEGG) pathways during different ripening stages.

**Additional file 7.** The expression and functional annotation of genes shown in Fig. 5b.

**Additional file 8.** The expression and functional annotation of genes shown in Fig. 6.

**Additional file 9.** Primer sequences used for real-time quantitative PCR.

## Data Availability

The datasets supporting the results of this article are included within the article and its additional files. All of the obtained sequences from apricot were deposited in the NCBI Sequence Read Archive (SRA) repository under accession number PRJNA530709 and were released on April 3, 2019.

## Abbreviations

AP2: Ethylene-responsive transcription factor AP2
bHLH: Basic helix-loop-helix protein
BHT: Butylhydroxytoluene
BR: Brassinosteroid
BZR1: BRASSINAZOLE RESISTANT1
CCD1/4: Carotenoid cleavage dioxygenase 1/4
CCI: Citrus color index
CCS: Capsanthin/capsorubin synthase
CHYB: β-carotene hydroxylase
CM: Commercial maturation
COP1-like: CONSTITUTIVE PHOTOMORPHOGENIC 1-like
CRTISO: Carotenoid isomerase
DEGs: Differentially expressed genes
DMAPP: Dimethylallyl diphosphate
DXS: Deoxy-d-xylulose-5-phosphate synthase
EBR: 2,4-epibrassinolide
ERF003/5/12: Ethylene-responsive transcription factor 003/5/12
FDR: False discovery rate
FPKM: Fragments per kilobase of transcript per million mapped reads
FR: Fully ripe
FW: Fresh weight
GGPP: Geranylgeranyl diphosphate
GGPPS: Geranylgeranyl diphosphate synthase
GLK1/2: GOLDEN 2-LIKE transcription factor
GO: Gene ontology
HPLC: High-pressure liquid chromatography
IPP: Isopentenyl diphosphate isomerase
KEGG: Kyoto Encyclopedia of Genes and Genomes
LCYB: β-cyclase
LCYE: ε-cyclase
LT: Luntaixiaobaixing
MADS14: MADS-box transcription factor 14
MEP: Methyl-D-erythritol 4-phosphate
MTBE: Methyl tert-butyl ether
MYB1R1/44: Transcription factor MYB1R1/44
MYC2: Transcription factor MYC2
NAC2/25: Transcription factor NAC2/25
NCED1: 9-cis-epoxycarotenoid dioxygenase 1
NXS: Neoxanthin synthase
PCA: Principal component analysis
PDS1: Phytoene desaturase 1
PIF3/4: Phytochrome interacting factor 3/4
PSY: Phytoene synthase
qRT-PCR: Quantitative real time reverse transcriptase PCR
RAP2–12: Ethylene-responsive transcription factor RAP2–12
RIN: Ripening inhibitor
RNA-Seq: RNA sequencing
SRA: Sequence Read Archive
T: Turning
TA: Titratable acidity
TSS: Total soluble solids
VDE1: Violaxanthinde-epoxidase 1
WGCNA: Weighted gene coexpression network analysis
WRKY6/31/69: Transcription factor WRKY6/31/69
ZDS: ζ-carotene desaturase
ZEP: Zeaxanthin epoxidase
Z-ISO: 15-cis-zeta-carotene isomerase.
