# Supplementary material for: Identification of key genes and regulators associated with carotenoid metabolism in apricot (Prunus armeniaca) fruit using weighted gene coexpression network analysis
Source: BMC Genomics. 2019 Nov 20;20:876. doi: 10.1186/s12864-019-6261-5 (PMC6865023; doi:10.1186/s12864-019-6261-5)

**Additional file 3 Gene ontology (GO) classification map.** The horizontal axis represents the type of GO function; The vertical axis on the right represents the number of Unigene annotated to the corresponding GO function; The vertical axis on the left represents the percentage of the number of Unigene in the total.


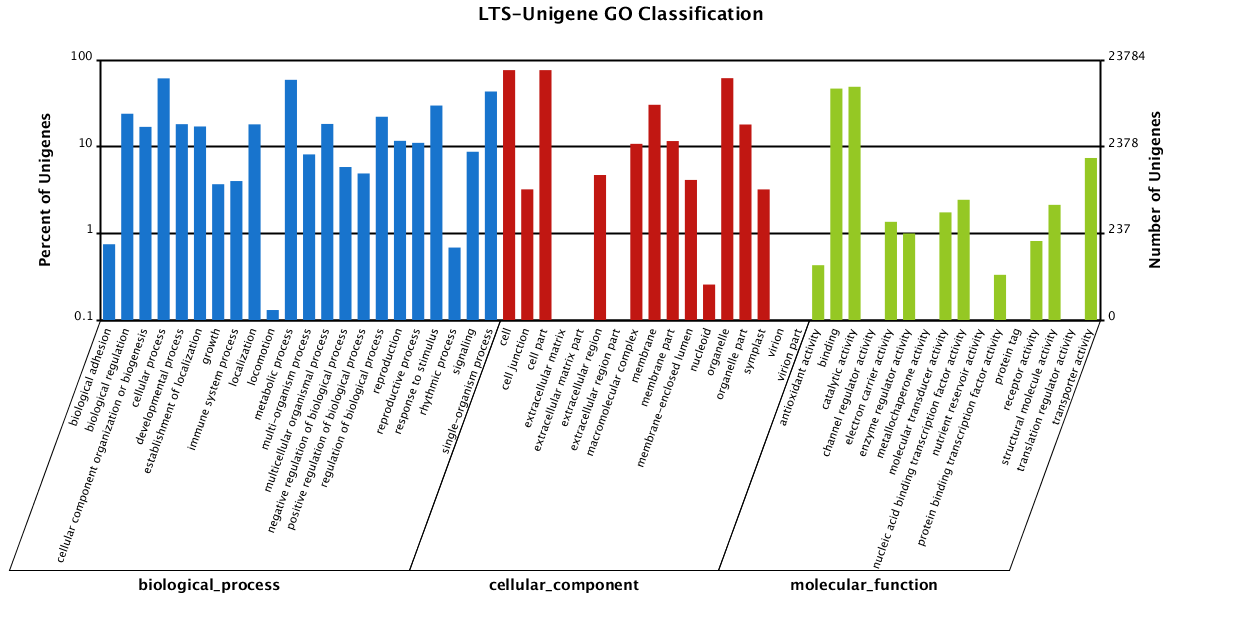

Supplement: Supplementary file 3 — Additional file 3. Gene ontology (GO) classification map. The horizontal axis represents the type of GO function; the vertical axis on the right represents the number of unigenes annotated to the corresponding GO function; and the vertical axis on the left represents the percentage of the number of unigenes within the total. [file 12864_2019_6261_MOESM3_ESM.docx]
