# Supplementary material for: Identification of key genes and regulators associated with carotenoid metabolism in apricot (Prunus armeniaca) fruit using weighted gene coexpression network analysis
Source: BMC Genomics. 2019 Nov 20;20:876. doi: 10.1186/s12864-019-6261-5 (PMC6865023; doi:10.1186/s12864-019-6261-5)

**Additional file 5** **Gene ontology (GO) annotation of differential expressed genes (DEGs).** (A) T vs CM, (B)T vs FR, (C) CM vs FR. Unigenes were annotated in three categories: biological process, cellular component and molecular functions. Right y-axis indicates the number of genes in a category; left y-axis indicates the percentage of a speciﬁc gene.


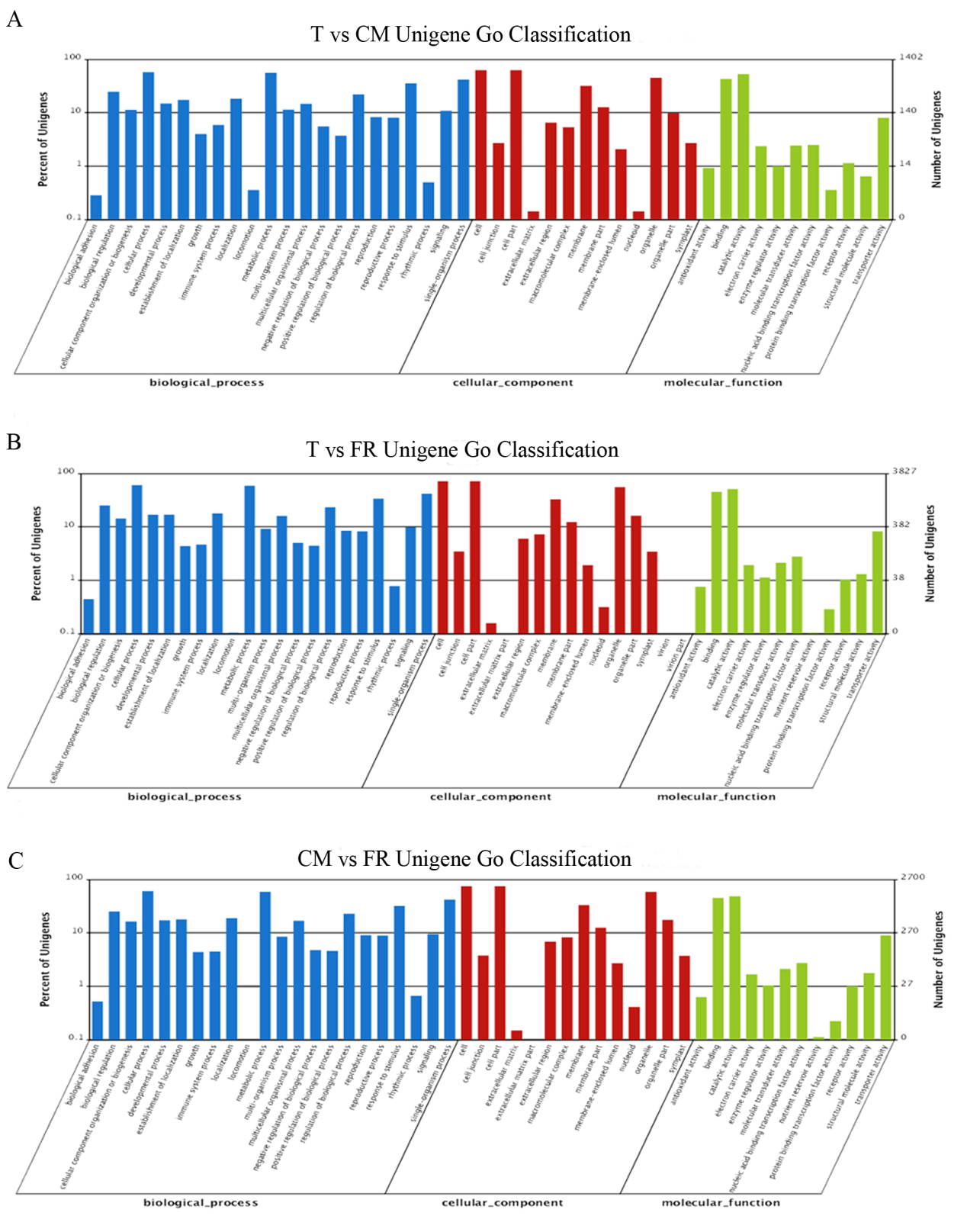

Supplement: Supplementary file 5 — Additional file 5. Gene ontology (GO) annotation of differentially expressed genes (DEGs). (A) T vs CM, (B) T vs FR, (C) CM vs FR. The unigenes were annotated in three categories: biological process, cellular component and molecular functions. The right y-axis indicates the number of genes in a category, and the left y-axis indicates the percentage of a specific gene. [file 12864_2019_6261_MOESM5_ESM.docx]
